# Ambulatory Blood Pressure Monitoring in Children and Adults Prenatally Exposed to Dexamethasone Treatment

**DOI:** 10.1210/clinem/dgac081

**Published:** 2022-02-11

**Authors:** Leif Karlsson, Lena Wallensteen, Anna Nordenström, Rafael T Krmar, Svetlana Lajic

**Affiliations:** 1 Department of Women’s and Children’s Health, Karolinska Institutet, Pediatric Endocrinology Unit, Karolinska University Hospital, Stockholm, Sweden; 2 Department of Physiology and Pharmacology, Biomedicum 5B, Karolinska Institutet, Stockholm, Sweden

**Keywords:** prenatal treatment, dexamethasone, congenital adrenal hyperplasia, blood pressure, ambulatory blood pressure monitoring, hypertension

## Abstract

**Context:**

The clinical use of dexamethasone (DEX) prenatally to reduce virilization of external genitalia in female fetuses with congenital adrenal hyperplasia (CAH) is efficient but still controversial. It remains challenging to prevent the excessive exposure of DEX in unborn healthy babies during the first trimester of pregnancy.

**Objective:**

Since endogenous glucocorticoids contribute to the maintenance of blood pressure (BP) and since events during fetal life may program the fetus and affect future metabolic health, the aim of this study was to analyze ambulatory BP measurements in CAH-unaffected children and adults that were prenatally exposed to DEX treatment.

**Methods:**

Ambulatory BP measurements were analyzed in 33 (16 female) DEX-treated participants aged 5.1 to 26.3 years (19 participants aged ≤ 18 years) and in 54 (28 female) age- and sex-matched apparently healthy controls aged 5.5 to 25.3 years (27 participants aged ≤ 18 years) with ambulatory normotension.

**Results:**

Participants’ age, height, weight, and body mass index were similar between the DEX-treated group and the control group. Heart rate, 24-hour BP, pulse pressure, and nighttime dipping did not statistically significantly differ between DEX-treated participants and controls.

**Conclusion:**

Our study suggests that prenatal DEX treatment in CAH-unaffected children and adults does not appear to adversely affect ambulatory BP later in life. Our observations need to be confirmed in larger studies.

In congenital adrenal hyperplasia (CAH), the majority of affected individuals display mutations in the 21-hydroxylase (*CYP21A2*) gene. *CYP21A2* gene mutations result in the reduced synthesis of cortisol and mineralocorticoid in the adrenal cortex and a subsequent overproduction of androgens (1). The increase in androgen production causes virilization of the external genitalia in female fetuses in early gestation that may require surgery depending on the severity of the virilization (1).

Since the 1980s, pregnant women at risk of having a child with CAH have been offered treatment with dexamethasone (DEX) to reduce virilization in affected female fetuses (2-5). Importantly, to achieve its clinical goal, the treatment should be initiated before gestational week (GW) 7, since external genitalia develop between GW 8 and 12. Because fetal genotyping is not possible at this time point in early gestation, it results in unnecessary prenatal exposure to DEX in CAH-unaffected children as well as boys with CAH (5). Tardy-Guidollet et al (5) have shown that early fetal sex typing (SRY detection) using cell-free fetal DNA from maternal blood is reliable and can be used to avoid unnecessary treatment in boys if performed after GW 4.5. However, preventing excessive exposure of DEX in healthy girls during the first trimester of pregnancy by sex typing remains challenging. Of note, the administration of DEX results in cortisol levels greater than normal physiological fetal glucocorticoid (GC) levels (1).

Cortisol serves a variety of important functions, including producing effects on the cardiovascular system and the kidneys that may give rise to systemic hypertension (6). Studies have shown negative effects due to early prenatal DEX treatment in the context of CAH in multiple biological functions (7-11). Adverse effects on glucose metabolism with an indication of affected β-cell function/mass (8, 9) and higher cholesterol levels in DEX-treated individuals have been observed (9). Moreover, effects on the central nervous system with regard to cognitive function, especially working memory (11-13) and altered brain structure (eg, an enlarged amygdala), have also been observed in follow-up studies (14). We have previously hypothesized that the observed adverse outcomes from prenatal DEX treatment is caused by altered fetal programming through epigenetic mechanisms (7). While a few of the relevant outcomes in prenatally exposed individuals have been investigated in follow-up studies, the long-term effects on postnatal blood pressure (BP) in CAH-unaffected children and adults prenatally treated with DEX are poorly understood. Presently, the use of ambulatory BP monitoring (ABPM) has been acknowledged by international guidelines as a key clinical tool in hypertension diagnosis both in children and in adults (15-18). This is based on data indicating that mean ambulatory BP values are a more sensitive risk predictor and better prognostic value than office BP for cardiovascular outcomes such as coronary morbid or fatal events and stroke (19).

Against this background, the aim of this study was to analyze ambulatory BP measurements in CAH-unaffected children and adults that were prenatally exposed to DEX treatment during the first trimester of fetal life.

## Materials and Methods

### Study Cohort

The present study includes CAH-unaffected participants that were prenatally exposed to DEX. As previously described, the participants were identified from all at-risk pregnancies that were treated with DEX in Sweden between 1984 and 2010 (PREDEX study) (9), mean age 15.7 years (median = 17.7, range = 5.1-26.3 years).

Age- and sex-matched apparently healthy controls were recruited from the Swedish Civil Registration System, all living in Stockholm County. Invitation letters were sent to 394 potential controls. Briefly, 232 individuals did not send any response, 62 declined participation, and 100 people agreed to participate in the larger PREDEX study. Of them, 70 participants underwent ABPM. To be included in the study, apparently healthy controls must have had absence of disease based on normal medical history, physical examination, and laboratory values within the normal reference range. Laboratory tests included blood status, glucose-insulin metabolism, renal function, and blood lipids; the results of this investigation are presented elsewhere (9).

Thirty-seven (18 females) DEX-treated participants and 70 (34 females) apparently healthy controls were included in this study. Data were analyzed separately for children and adults, that is, participants younger than or equal to 18 years and those older than 18 years, respectively.

The Regional Ethics Committee of Stockholm (Nos. 99-153 and 2011/1764-32) approved the study and all participants and parents of children younger than 18 years gave their informed consent before study inclusion.

### Ambulatory Blood Pressure Monitoring

As previously described (20, 21), ABPM was performed using an oscillometric device SpaceLabs 90207 (SpaceLabs Medical Inc), which has been validated both in children and adults (22, 23). BP was recorded on the nondominant arm, using an appropriate-sized arm cuff. Since the present study was conducted for research purposes only, the device was programmed for cuff insufflations every 30 minutes from 7 am to 10 pm and every 60 minutes from 10 pm to 7 am to minimize sleep disturbances (24). Participants were instructed to keep a diary of daily activities during the ABPM measurement. Nighttime was defined according to the period of nighttime sleep based on participants’ diaries. Valid ABPM recordings were regarded as those having at least 70% of the expected readings available.

The ABPM results included mean 24-hour, daytime and nighttime systolic BP, diastolic BP, mean arterial pressure, and heart rate. Pulse pressure was calculated by the difference between mean systolic BP and mean diastolic BP. Blood pressure dipping, that is, the nocturnal fall in systolic or diastolic BP, was computed by subtracting the mean nighttime ambulatory BP from the mean daytime ambulatory BP.

Ambulatory normotension for participants younger than or equal to 18 years was defined as systolic and diastolic BP less than the age-, sex-, and height-specific 95th percentile for daytime and nighttime BP values as per pediatric ABPM normative data (15, 22). This definition of ambulatory normotension in children and adolescents was valid only if the ambulatory BP values were below the BP thresholds for defining ambulatory normotension in participants older than 18 years, that is, 24-hour 130/80 mm Hg; daytime 135/85 mm Hg, and nighttime 125/75 mm Hg, respectively (17).

### Statistical Analyses

Results are reported as mean ± SD, unless otherwise indicated. ABPM results are presented as raw data. In participants younger than or equal to 18 years, the BP data were also normalized to SD scores (SDSs) by applying the least mean square method as first described by Cole and Green (25) and adapted by Wühl et al (22). This linear transformation represents the distance in units of SD between a given individual’s mean ambulatory BP values and the mean reference ambulatory BP values for a given sex and height provided in the revised version of the original pediatric normative ABPM data (26). Thus, by applying this method, the data analysis in children and adolescents for both sexes and all heights can be combined.

All variables were tested for normality using the Shapiro-Wilk test; homogeneity of variance was tested with the Levene test. Several parameters were not normally distributed, but all showed equal variance. Accordingly, multiple linear regression statistics was chosen to evaluate the difference in mean BP values. Each model corrected for participant body mass index and included participant sex to detect sex-specific effects from prenatal DEX treatment.

Participants in the control group diagnosed as having ambulatory hypertension were excluded from the analysis of ABPM results. The presence of ambulatory hypertension in DEX-treated participants and in apparently healthy controls was analyzed by using the Fisher exact test.

All statistical analyses were performed in R software, version 4.0.2 (http://www.Rproject.org), and a 2-tailed α level of *P* less than .05 was used to define statistical significance for all comparisons (27). We did not control for multiple comparisons to avoid missing small but potentially clinically relevant effects. Effect sizes were calculated, where applicable, as Cohen’s *d* so that positive effect sizes represent better performance in controls (28). For Cohen’s *d*, effects are categorized as large when *d* was greater than or equal to 0.80, moderate when *d* was greater than or equal to 0.50, and small when *d* was greater than or equal to 0.20 (28). Given our sample size, using an α level of 0.05 and assuming a power level of 80% for our analysis, the estimated absolute minimum detectable effect size is 0.86 for participants younger than or equal to 18 years and 0.95 for participants older than 18 years.

## Results

Nine patients in this study were excluded from the analysis because their ABPM were incomplete (4 DEX-treated participants and 5 apparently healthy controls, respectively). In addition, 11 apparently healthy controls (6 aged ≤ 18 and 5 aged > 18 years) diagnosed as having ambulatory hypertension were excluded from the statistical analysis of ABPM results. The presence of daytime and nighttime hypertension is described in detail both for DEX-treated participants and apparently healthy controls in Table 1. The presence of diagnosed hypertension did not statistically significantly differ between DEX-treated and control groups (*P* > 0.05), for participants younger than or equal to 18 years and those older than 18 years; see Table 1.

**Table 1.** Overview of systolic and diastolic hypertension in dexamethasone-treated participants and controls

	DEX (total n = 5)	Control (total n = 11)
**Participants ≤ 18 y**		
Daytime SHT	–	1^*a*^
Daytime SHT and DHT	–	1^*a*^
Nighttime SHT	2	–
Nighttime DHT	2	3
Daytime SHT and DHT Nighttime DHT	–	1
**Participants > 18 y**		
Daytime SHT	–	1^*a*^
Daytime DHT	–	1^*a*^
Nighttime DHT	–	1
Daytime and nighttime SHT	1^*a*^	1^*a*^
Daytime and nighttime SHT Nighttime DHT	–	1

Abbreviations: DEX, dexamethasone; DHT, diastolic hypertension; SHT, systolic hypertension.

^*a*^Mean 24-hour blood pressure was in the hypertensive range.

On comparing the characteristics of the groups, neither age, length, weight, nor body mass index differed statistically significantly between DEX-treated participants and controls for participants younger than or equal to 18 or those older than 18 years; Table 2. All participants were younger than 30 years when the study was conducted. Characteristics of study participants are summarized in Table 2.

**Table 2.** Group averages (mean ± 1 SD) and test statistics for participants’ age, height, weight, and body mass index

			DEX			DEX × sex		
	DEX (M ± SD)	Controls (M ± SD)	β	*t* stat	*P*	β	*t* stat	*P*
**Participants ≤ 18 y**								
No., female	19 (9)	27 (11)						
Age, y	10.9 (4.7)	12.9 (3.9)	0.6	0.2	.865	–1.9	–0.8	.440
Height, cm	141.8 (23.6)	153.2 (18.7)	16.5	0.9	.378	–19.7	–1.6	.112
Weight, kg	37.2 (17.3)	45.6 (17.2)	18.4	1.3	.213	–19.1	–2.0	.052
BMI	17.2 (2.8)	18.7 (3.8)	3.1	1.1	.274	–3.3	–1.8	.078
**Participants > 18 y**								
No., female	14 (7)	27 (17)						
Age, y	21.8 (2.2)	21.2 (1.9)	0.1	0.0	.968	0.3	0.2	.809
Height, cm	173.8 (9.2)	173.1 (8.9)	–3.4	–0.5	.603	1.4	0.4	.717
Weight, kg	74 (11.3)	69 (14.5)	2.2	0.2	.866	0.7	0.1	.931
BMI	24.6 (4.4)	22.9 (4.0)	1.3	0.3	.783	0.2	0.1	.949

Abbreviations: BMI, body mass index; DEX, dexamethasone; M, mean.

For participants younger than or equal to 18 years and those older than 18 years, no statistically significant differences were observed in any of the measures obtained from 24-hour BP monitoring between DEX-treated individuals and apparently healthy controls. The results of these comparisons are presented in Tables 3 and 4. Regarding the analysis of SDSs in participants younger than or equal to 18 years, no differences were observed on comparing SDSs between treated DEX-treated individuals and apparently healthy controls. Analysis of SDSs is presented in Table 5.

**Table 3.** Group averages (M ± 1 SD) for all measures in the age group younger than or equal to 18 years

			DEX				DEX × sex		
	n = 19	n = 27	β	*d*	*t* stat	*P*	β	*t* stat	*P*
Pulse pressure, 24-h	45.1 (7.8)	44.1 (5.8)	2.9	0.1	0.5	.642	–0.5	–0.1	.906
Pulse pressure, day	45.1 (8.4)	44.1 (5.9)	1.2	0.1	0.2	.846	0.7	0.2	.864
Pulse pressure, night	45.1 (7.8)	43.8 (6.2)	6.3	0.2	1.0	.316	–2.7	–0.6	.526
SBP, 24-h	110.1 (7.9)	110.7 (7.1)	2.0	–0.1	0.3	.780	–1.5	–0.3	.751
SBP, daytime	114.2 (8.5)	114.3 (7.2)	2.1	0.0	0.3	.775	–1.3	–0.3	.791
SBP, nighttime	102.1 (7.8)	101.1 (7.6)	6.7	0.1	0.9	.361	–3.5	–0.7	.470
DBP, 24-h	64.9 (3.8)	66.6 (4.0)	–0.9	–0.4	–0.3	.801	–1.0	–0.4	.672
DBP, daytime	69.2 (4.7)	70.1 (4.1)	0.8	–0.2	0.2	.815	–2.0	–0.9	.398
DBP, nighttime	57.0 (5.1)	57.3 (4.4)	0.3	–0.1	0.1	.941	–0.9	–0.3	.772
MAP, 24-h	81.1 (4.0)	81.9 (4.3)	2.1	–0.2	0.6	.584	–2.3	–0.9	.383
MAP, daytime	84.6 (4.9)	85.0 (4.3)	4.0	–0.1	1.0	.319	–3.4	–1.3	.207
MAP, nighttime)	74.0 (4.7)	73.9 (4.9)	3.6	0.0	0.8	.424	–2.4	–0.8	.419
SBP nighttime dipping, %	10.6 (4.3)	11.5 (4.7)	–4.7	–0.2	–1.1	.269	2.4	0.8	.403
DBP nighttime dipping, %	17.4 (7.6)	18.1 (5.4)	0.5	–0.1	0.9	.932	–1.3	–0.3	.754
Heart rate (24-h)	83.6 (10.7)	81.3 (9.4)	8.8	0.2	1.0	.301	–6.4	–1.1	.261
Heart rate, day	87.2 (12.9)	85.2 (9.9)	8.4	0.2	0.9	.358	–6.7	–1.1	.277
Heart rate, night	76.3 (9.8)	71.0 (9.5)	10.5	0.6	1.2	.229	–5.1	–0.9	.381

β Coefficient values, Cohen *d*, *t* statistics, and *P* values for the main effect of DEX and the interaction between DEX and sex are presented. Controls exhibiting 24-hour, daytime or nighttime systolic and/or diastolic hypertension are excluded. No statistically significant between-group differences were observed.

Abbreviations: DBP, diastolic blood pressure; DEX, dexamethasone; M, mean; MAP, mean arterial pressure; SBP, systolic blood pressure.

**Table 4.** Group averages (M ± 1 SD) for all measures in the age group older than 18 years

			DEX				DEX × sex		
	n = 14	n = 27	β	*d*	*t* stat	*P*	β	*t* stat	*P*
Pulse pressure, 24-h	49.4 (7.4)	46.4 (5.6)	9.1	0.5	1.5	.149	–4.2	–1.1	.272
Pulse pressure, day	50.0 (7.4)	46.5 (6.1)	9.8	0.5	1.5	.137	–4.3	–1.1	.279
Pulse pressure, night	48.3 (7.7)	46 (5.1)	7.4	0.4	1.1	.263	–3.6	–0.9	.378
SBP, 24-h	118.7 (7.3)	113.8 (7.4)	12.7	0.7	1.7	.107	–5.7	–1.2	.238
SBP, daytime	123.1 (7.2)	118.2 (8.2)	9.1	0.6	1.1	.279	–3.3	–0.6	.521
SBP, nighttime	107.9 (8.1)	102.6 (7.6)	13.5	0.7	1.6	.109	–6.0	–1.2	.245
DBP, 24-h	69.3 (4.9)	67.4 (6.2)	3.6	0.3	0.6	.563	–1.4	–0.4	.708
DBP, daytime	73.1 (5.7)	71.7 (6.7)	–0.7	0.2	–0.1	.925	1.0	0.2	.811
DBP, nighttime	59.6 (4.2)	56.7 (6.2)	6.1	0.5	1.0	.302	–2.4	–0.7	.507
MAP, 24-h	85.9 (4.1)	83.5 (5.5)	4.6	0.5	0.8	.405	–1.9	–0.6	.572
MAP, daytime	89.7 (5.1)	87.3 (6.0)	1.5	0.4	0.2	.813	0.2	0.0	.966
MAP, nighttime	76.1 (3.9)	73.6 (6.0)	5.2	0.5	0.9	.355	–2.4	–0.7	.493
SBP nighttime dipping, %	12.3 (4.4)	13.0 (6.7)	–3.9	–0.1	–0.6	.562	2.2	0.5	0.591
DBP nighttime dipping, %	18.2 (5.9)	20.7 (8.0)	–9.1	–0.3	–1.1	.269	4.4	0.9	.380
Heart rate, 24-h	72.3 (10.6)	73.7 (10.5)	–9.0	–0.1	–0.9	.360	4.5	0.8	.456
Heart rate, day	75.8 (12.0)	77.6 (10.9)	–14.3	–0.2	–1.3	.198	7.6	1.1	.263
Heart rate, night	63.8 (11.1)	64.5 (12.9)	–11.1	–0.1	–1.0	.344	6.0	0.8	.402

β Coefficient values, Cohen *d*, *t* statistics, and *P* values for the main effect of DEX and the interaction between DEX and sex are presented. Controls exhibiting 24-hour, daytime or nighttime systolic and/or diastolic hypertension are excluded. No statistically significant between-group differences were observed.

Abbreviations: DBP, diastolic blood pressure; DEX, dexamethasone; M, mean; MAP, mean arterial pressure; SBP, systolic blood pressure; SDS, SD scores.

**Table 5.** Group averages (M ± 1 SD) for measures SD score–transformed in the age group less than or equal to 18 years

			DEX			
	n = 19	n = 27	β	*d*	t-stat	*P*
SBP SDS, 24-h	0.1 (1.1)	–0.2 (1)	0.1	0.3	0.3	.789
SBP SDS, daytime	0 (1.1)	–0.3 (1.1)	0.1	0.3	0.3	.757
SBP SDS, nighttime	0.4 (1)	0 (0.9)	0.3	0.4	0.9	.356
DBP SDS (24-h)	–0.3 (0.7)	–0.1 (0.8)	–0.4	–0.3	–1.7	.103
DBP SDS, daytime	–0.5 (0.8)	–0.5 (0.8)	–0.3	0.0	–1.4	.167
DBP SDS, nighttime	0.3 (0.9)	0.3 (0.7)	–0.1	0.0	–0.4	.692
MAP SDS, 24-h	0.2 (0.7)	0.1 (0.8)	–0.1	0.1	–0.6	.578
MAP SDS, daytime	–0.1 (0.8)	–0.2 (0.8)	–0.1	0.1	–0.5	.603
MAP SDS, nighttime	0.6 (0.8)	0.5 (0.7)	0.0	0.1	–0.1	.959

β Coefficient values, Cohen *d*, *t* statistics, and *P* values for the main effect of DEX and the interaction between DEX and sex are presented. Controls exhibiting 24-hour, daytime or nighttime systolic and/or diastolic hypertension are excluded. No statistically significant between-group differences were observed.

Abbreviations: DBP, diastolic blood pressure; DEX, dexamethasone; M, mean; MAP, mean arterial pressure; SBP, systolic blood pressure; SDS, SD scores.

## Discussion

We analyzed ambulatory BP measurements in children and adults at risk of having CAH and that were prenatally treated with DEX. We did not observe any differences in ambulatory BP values between DEX-treated participants and apparently healthy normotensive controls in the age interval studied, that is, younger than 30 years. Our study suggests that prenatal DEX treatment during early fetal life does not appear to have long-term adverse effects on BP later in life.

Previous studies of late-gestation GC exposure in humans and the possible effect on postnatal BP have shown conflicting results (29-37). Several follow-up studies found no effect on BP in children or young adults (29-34). However, some investigations reported increased aortic stiffness, which may precede the occurrence of sustained hypertension (35-37). Studies assessing the effect on the hypothalamic-pituitary-adrenal axis have primarily evaluated the effects at early age post partum and during childhood. The hypothalamic-pituitary-adrenal axis appears sensitized with an increased cortisol secretion after stress exposure by late prenatal GC treatment and in the case of second to third trimester maternal stress (38-40). However, the effect of daily exposure for approximately 6 weeks in early gestation might be even more detrimental for the fetus and the growing child (41). Animal studies on sheep have shown higher BP in offspring exposed to early DEX treatment (42-46). There are also animal studies indicating a possible causal relationship between prenatal exposure to GCs and the occurrence of postnatally reduced number of nephrons and increased reactivity in the arterioles (45, 47, 48). The latter findings may contribute to the pathogenesis of hypertension (6, 49, 50).

The present study adds additional information regarding the long-term safety of prenatal DEX treatment investigated by our group. There are results suggesting that early imprinting of the fetus can take place in the context of prenatal DEX treatment for CAH, which may adversely affect postnatal health (9, 11, 14). We have observed higher glucose levels, higher cholesterol levels, impaired cognition, and larger amygdala size in this cohort of patients (9, 11, 14). These findings were partially confirmed by Riveline et al (8), indicating that adults treated during the first trimester with DEX had statistically significantly lower insulin secretion compared with controls.

We have also observed epigenetic alterations of DNA that may mediate a mechanism by which prenatal DEX treatment could affect outcomes (7). These results showed that differences in DNA methylation, measured in peripheral CD4^+^ T cells, were observed between controls and treated cases. The differences were enriched within regions of the genome associated with immune function and inflammation (7). Epigenetic changes associated with DEX were also found in the *CYP11B2* gene, which codes for aldosterone synthase, an enzyme involved in aldosterone synthesis (14). In addition, methylation at a CpG site in the *FKBP5* gene was associated with increased radial diffusivity in the white matter in the brain in adults treated prenatally with DEX, providing further evidence supporting our line of reasoning (14). In our study, we did not observe abnormal ambulatory BP, pulse BP, or heart rate patterns in this relatively young cohort. Nonetheless, on the larger scope, with the existing evidence on the negative metabolic and cognitive effects observed in individuals treated during the first trimester (8) and the epigenetic differences identified in our cohort (7), concerns regarding the long-term safety of prenatal DEX treatment of CAH remain.

### Limitations

The sample size of our cohort study of CAH-unaffected children and adults prenatally treated with DEX is relatively small. The age range in our study cohort is relatively young, because prenatal DEX treatment has been performed only since the 1980s. This may be an important limitation since the prevalence of hypertension increases with age. Future studies should therefore be conducted in older individuals and larger cohort studies. Another limitation is the lack of office BP measurements. Consequently, we were not able to identify 4 major BP phenotypes, namely sustained normotension (presence both of office and ambulatory normotension), sustained hypertension (presence both of office and ambulatory hypertension), white-coat hypertension (presence of office hypertension and ambulatory normotension), and masked hypertension (presence of office normotension and ambulatory hypertension) (15-18). Finally, 16% of the apparently healthy controls were diagnosed as having ambulatory hypertension. Although this figure appears to be lower than the prevalence of hypertension reported among adults aged 18 to 39 years (51), we have no good explanation for this observation.

### Conclusion

Our findings suggest that, when compared to apparently healthy normotensive controls, prenatal exposure to DEX in CAH-unaffected children and adults does not translate into higher ambulatory BP values in this relatively young cohort. Our results are preliminary and need to be confirmed in larger studies and over a longer period of time. Finally, regardless of the absence of effects on BP after early prenatal DEX treatment in CAH-unaffected children and young adults, there are still concerns regarding their long-term health.

## Data Availability

Some or all data sets generated during and/or analyzed during the present study are not publicly available but are available from the corresponding author on reasonable request.

## Abbreviations

ABPM: ambulatory blood pressure monitoring
BP: blood pressure
CAH: congenital adrenal hyperplasia
*CYP21A2*: 21-hydroxylase gene
DEX: dexamethasone
GC: glucocorticoid
GW: gestational week
